# Remote activities of cognitive stimulation for older adults during the COVID-19 pandemic: a systematic review

**DOI:** 10.1590/0102-311XEN081923

**Published:** 2024-02-26

**Authors:** Etiene Souza Madeira, Priscilla Alfradique de Souza, Anderson Amaral

**Affiliations:** 1 Programa de Pós-graduação em Enfermagem, Universidade Federal do Estado do Rio de Janeiro, Rio de Janeiro, Brasil.

**Keywords:** Telerehabilitation, Cognition, Elderly, COVID-19, Telerreabilitação, Cognição, Idoso, COVID-19, Telerrehabilitación, Cognición, Anciano, COVID-19

## Abstract

Cognitive stimulation activities for older adults are generally carried out in face-to-face workshops. However, during the COVID-19 pandemic, these activities and consultations became remote due to social isolation, enabling care to continue safely. This study aims to analyze the remote cognitive stimulation and/or telerehabilitation activities for older people that were carried out as an intervention during the COVID-19 pandemic. This is a systematic review study with five selected articles, conducted according to the PRISMA statement methodology. Among the main results, the feasibility and acceptance of remote cognitive stimulation activities using technologies during the pandemic stand out, reflecting on future and expanded use for different realities and cultures. the studies reviewed also indicate the stabilization and improvement of the cognitive state and of depressive and anxious feelings, as well as the maintenance of independence of these participants, with an increase in scores on scales applied before and after the interventions. In conclusion, the activities carried out in cognitive stimulation and/or telerehabilitation therapies for older adults as an intervention during the COVID-19 pandemic had an average of 47 participants; the technologies used for the activities were tablet and personal computer; pre-installed programs were the most used strategy; and the interventions lasted from 1 to 3 months, with activities 2 to 3 times per week. The reinvention of techniques aimed at stimulating and rehabilitating the cognitive health of the older adults, via technologies as a strategy to replace or complement face-to-face activities, promotes the cognitive and mental health and independence of the older population.

## Resumo

As atividades de estímulo cognitivo em idosos são geralmente realizadas por meio de oficinas presenciais; porém durante a pandemia da COVID-19, com o isolamento social, as atividades e consultas passaram a ser remotas para não interromper os cuidados. O objetivo deste estudo é analisar as atividades de estímulo cognitivo remoto e/ou telerreabilitação em idosos como intervenção durante a pandemia da COVID-19. Esta é uma revisão sistemática de cinco artigos selecionados, conduzida segundo a metodologia PRISMA. Os principais resultados incluem a viabilidade e aceitação de atividades de estímulo cognitivo remoto utilizando tecnologias durante a pandemia, refletindo em uso futuro e ampliado para diferentes realidades e culturas. Estudos também apontam para a estabilização e melhoria do estado cognitivo, dos sentimentos de depressão e ansiedade e da manutenção da independência desses participantes, com aumento nas pontuações nas escalas aplicadas depois das intervenções. Conclui-se que as atividades realizadas em terapias de estímulo cognitivo e/ou telerreabilitação em idosos como intervenção durante a pandemia da COVID-19 tiveram em média 47 participantes, os dispositivos tecnológicos utilizados foram o tablet e o computador, os programas pré-instalados foram a estratégia mais utilizada, com duração da intervenção de 1 a 3 meses e atividades realizadas 2 a 3 vezes por semana. A reinvenção de técnicas que visam estimular e reabilitar a saúde cognitiva dos idosos, incluindo o uso de tecnologias como estratégia para substituir ou complementar as atividades presenciais, promove a saúde cognitiva e mental e a independência dos idosos.

## Resumen

Las actividades de estimulación cognitiva en ancianos generalmente se realizan mediante talleres presenciales; pero, durante la pandemia de COVID-19, con el aislamiento social, las actividades y consultas se volvieron remotas para no interrumpir los cuidados. El objetivo de este estudio es analizar las actividades de estimulación cognitiva remota o telerrehabilitación en ancianos como intervención durante la pandemia de COVID-19. Se trata de una revisión sistemática de cinco artículos seleccionados, realizada según la metodología PRISMA. Los principales resultados incluyen la viabilidad y aceptación de actividades de estimulación cognitiva remota utilizando tecnologías durante la pandemia, lo que refleja un uso futuro y ampliado para diferentes realidades y culturas. Los estudios también apuntan a la estabilización y mejora del estado cognitivo, de los sentimientos de depresión y ansiedad y de la conservación de la independencia de estos participantes, con un aumento en las puntuaciones en las escalas aplicadas tras las intervenciones. Se concluye que las actividades realizadas en terapias de estimulación cognitiva o telerrehabilitación en ancianos como intervención durante la pandemia de COVID-19 tuvieron un promedio de 47 participantes, los dispositivos tecnológicos utilizados fueron la tableta y la computadora, los programas preinstalados fueron la estrategia más utilizada, con una duración de la intervención de 1 a 3 meses y actividades realizadas de 2 a 3 veces por semana. La reinvención de técnicas que tienen como objetivo estimular y rehabilitar la salud cognitiva de los ancianos, incluido el uso de tecnologías como estrategia para reemplazar o complementar las actividades presenciales, promueve la salud cognitiva y mental y la independencia de los ancianos.

## Introduction

The advance of science over the years, with improvements in health care and investments in prevention, has allowed the older adult population to grow. Brazil gained 4.8 million older adults in 2012, with a total of 23.5 million - 11% of the population that year -, with an annual growth of more than 4% ever since. In 2020, older adults represented 14% of the population, that is, 30 million. As a result, concern about age-related diseases became a central agenda in gerontological care, especially in relation to illnesses linked to memory and cognition, which are essential skills for maintaining autonomy and independence ^1^.

With aging, chronic degenerative conditions develop and have a major impact on the health-disease process of older adults. According to the American Psychiatric Association, the occurrence of severe damage and loss compromises functional performance by altering the noble functions of the central nervous system, in processes such as cortical atrophy and vascular alterations, leads to pathological aging. The main causes of this form of aging are chronic neurodegenerative diseases, mainly mild cognitive impairment (MCI) and dementia, currently classified by the *Diagnostic and Statistical Manual of Mental Disorders* - fifth edition (DSM-V), in the appropriate order, as mild neurocognitive disorder and major neurocognitive disorder (MND), especially the Alzheimer’s disease subtype ^2^ ^,^ ^3^.

Cognitive stimulation activities are used to prevent, promote and rehabilitate cognitive functions. This strategy maintains cognitive and memory stimulation and also promotes socialization among older adults. However, during the COVID-19 pandemic, the most affected population was older adults, due to social isolation and stricter restriction measures.

During the pandemic, previously used resources, such as workshops, consultations and essential follow-ups for the physical and mental health of older adults, had to be reformulated and adapted to the new context. One of the strategies used to enable this adaptation was to carry out these activities remotely. Technologies such as cell phones, smartphones and computers were used as tools for getting closer, socializing and returning to previous activities. This research aims to analyze the activities carried out in cognitive stimulation therapies and/or telerehabilitation for older adults as an intervention during the COVID-19 pandemic.

## Method

This is a systematic review study, conducted according to the *Preferred Reporting Items for Systematic Reviews and Meta-Analyses* (PRISMA statement) methodology ^4^.

This systematic review aims to gather evidence that fits predetermined eligibility criteria to answer a specific research question, using explicit and systematic methods that were selected to minimize bias, helping to make decisions based on more reliable data ^5^.

This systematic review has its protocol registered in the OSF Registries database (https://osf.io/mn267).

The PICO strategy, which stands for Patient, Intervention, Comparison and Outcomes, was used to draft the research question:

P - Older adults without cognitive impairment to those with moderate cognitive impairment.

I - Remote cognitive stimulation activities during the pandemic.

C - Older adults in social isolation without telerehabilitation interventions and/or cognitive stimulation.

O - Description of activities and positive results (improvement or stabilization of the cognitive pattern).

The research question was: “How have remote cognitive stimulation and/or telerehabilitation activities developed in older adults as an intervention to replace and/or complement face-to-face activities during the COVID-19 pandemic?”.

The inclusion criteria were: articles that address remote cognitive stimulation activities via intervention studies, with a time frame (Januray 1st, 2020 to July 1st, 2022) that encompasses the period of the COVID-19 pandemic, that include older adults (60 years or older) without cognitive impairment to moderate cognitive impairment, and that are written in Portuguese and English. The following types of article were excluded: case reports, expert opinions, integrative reviews, and articles without clear methodology.

The search was carried out in the CINAHL, Embase, Scopus and Web of Science (via CAPES Journals Portal) databases, from October 15 to 30, 2022, using the descriptors: *Telerehabilitation*; *Cognition*; *Elderly*; *COVID 19*; and the noncontrolled term *Cognitive stimulation therapy*.

**Figure 1:**
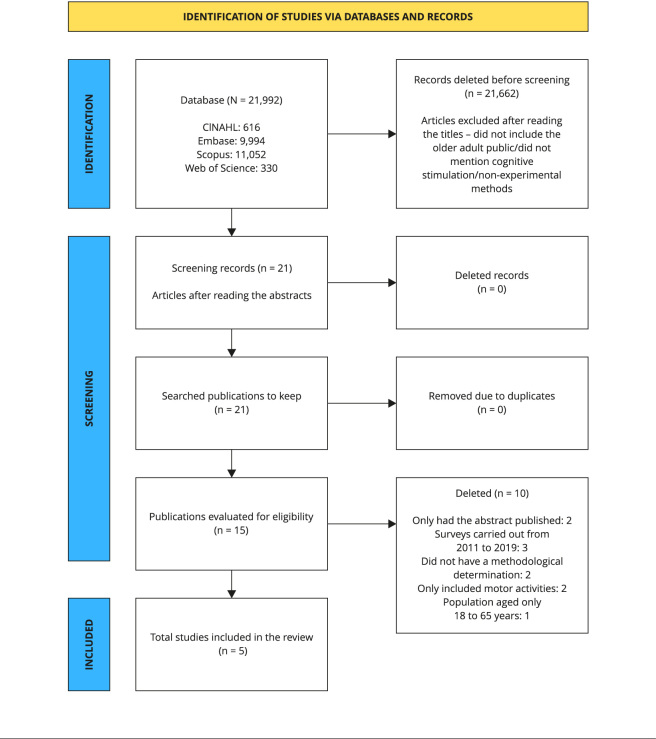
Flowchart of the identification and selection of articles for the systematic review.

The following Boolean operators were used: *Telerehabilitation AND Cognition AND Elderly*; *Cognitive stimulation therapy AND COVID 19*; *Cognitive stimulation therapy OR COVID*; *Telerehabilitation AND COVID 19* (Figure 1).

To collect data from the articles, a standard data instrument was created containing the following information: title, author, year, journal, country of publication, method, level of evidence, objective, sample size (number of participants), public, technology used, accessibility of the technique used for the older adults, frequency of stimulation (day/week), duration of cognitive stimulation (time per activity), research period (number of months the activity was carried with that population), description of the activities carried out, and results/conclusions.

The articles found were analyzed descriptively in stages by two researchers, who selected the articles. First, the sample was characterized using simple descriptive statistics and frequency distribution, with year of publication, language (Portuguese, English, and Spanish), methodology of the remote stimulation intervention, journal in which the article was published, and article database. Then, an exhaustive reading was carried out focusing on remote cognitive stimulation intervention activities using the technologies available during the COVID-19 pandemic and their results, organizing the articles in a table to facilitate the understanding of the data collected from each article.

As this study is a literature review and did not involve human beings, there was no need for approval by the Research Ethics Committee, as recommended by *Resolution n. 466/2012* of the Brazilian National Health Council.

## Results

According to the characterization of the selected studies, the distribution of publications by country was as follows: China, Switzerland, Brazil, Spain, and Italy. One study was carried out in each of these locations, that is, each one of them comprised 20% of the study sample. For year of publication, the following distribution was presented: 2020 (three studies - 60%), 2021 (one study - 20%), and 2022 (one study - 20%).

The sample studies were selected from the following databases: Scopus (one study - 20%), Embase (two studies - 40%), CINAHL (one study - 20%), and Web of Science (one study - 20%).

The methodologies of the selected studies were: case series of mixed methods (feasibility study) (one study - 20%), randomized controlled trial (three studies - 60%), and descriptive/exploratory (one study - 20%).

The included studies had a quantitative approach, with grades of recommendation A and B according to the Oxford Centre for Evidence-Based Medicine ^6^, with the following distribution: level of evidence A/1B (three studies - 60%) and level of evidence B/2C (two studies - 40%).

Box 1 shows the five articles included in this review, referring to interventions, remote cognitive stimulation activities with older adults that were carried out remotely during the COVID-19 pandemic and showed positive results in the improvement or stabilization of cognitive function or feasibility of cognitive stimulation activities for carrying out remotely in 100% of the studies.

**Box 1:** Articles included in the systematic review.

STUDY (YEAR)	COUNTRY/DATABASE/JOURNAL	EVIDENCE LEVEL	GENERAL OBJECTIVE	METHOD	RESULTS/CONCLUSIONS
Hui et al. ^8^ (2022)	China Scopus *Geriatric Nursing*	2C	To translate and culturally adapt V-iCST for the Hong Kong Chinese population; to assess the feasibility and acceptability of a virtual and 14-session version of the V-iCST program in Hong Kong	Feasibility study: mixed methods case series	Positive adherence, with all participants completing the 14 V-iCST sessions, indicating acceptable V-iCST. Most of them evaluated the activities as “liked it very much” (76.2%) or “liked it a little”. Reliable improvements were observed in cognition, quality of life, communication and/or mood for most participants. More than half of the participants maintained or improved their results in the MoCA 5 minutes and V-iCST (ADAS-Cog) intervention group. Four participants had clinically significant changes in depression. Six participants maintained or improved their MoCA 5 minutes scores. On the ADAS-Cog, four participants’ scores improved, with a reliable 11-point increase. Qualitative results: verbalized by participants. Convenience: the participants mentioned that it was convenient to take part in a virtual program, as it saves time, since they did not have to leave their homes
Manenti et al. ^9^ (2020)	Switzerland Embase *Frontiers in Aging Neuroscience*	1B	To evaluate the effectiveness of face-to-face VRRS system and compare it to the usual face-to-face cognitive treatment for individuals with MCI	Randomized clinical trial	The mean age of the participants was 76.5 years. In the comparison between the clinical-VRRS group that received telerehabilitation and the clinic-TAU group (cognitive face-to-face treatment as usual), a significant difference was observed in the clinical-VRRS group. The VRRS-clinic was more efficient than the clinic-TAU, improving memory, language, attention and vasoconstrictive skills. The at-home treatment showed that VRRS cognitive telerehabilitation has effects comparable to conventional rehabilitation in improving the cognitive abilities of patients with neurodegenerative diseases
Marinho et al. ^10^ (2021)	Brazil Embase *International Journal of Geriatric Psychiatry*	1B	To explore the feasibility and obtain preliminary data on the effectiveness of the CST-Brazil in a sample of 47 people with mild to moderate dementia treated at an outpatient unit	Blinded randomized controlled trial	Depression: the virtual group had lower scores for depression, while the face-to-face group experienced an increase in depressive symptoms. Activities of daily living: in the virtual group, there was a tendency towards an increase in independence, while in the face-to-face group, there were no significant changes. Intention to treat: it was positive, and the participants mentioned that they were interested in taking part in the activities. Severity of dementia: the results were small, positively suggesting a higher quality of life in participants with moderate dementia in the control group and no differences in the intervention group. Quality of life and caregiver burden were not significant main effects. Educational level: in caregiver burden, there was a tendency of time vs. educational level interaction that suggested a decrease in burden over time for participants with a lower level of education, but no changes in the group with a higher level of education
Goodman-Casanova et al. ^12^ (2020)	Spain CINAHL *Journal of Medical Internet Research*	2C	To explore the impact of confinement on the health and well-being of community-dwelling older adults with mild cognitive impairment or mild dementia	Exploratory	The health status of the participants was considered excellent, 97% of them had no symptoms of COVID-19. In total, 61% of the respondents reported general well-being and 70% maintained sleep quality. Negative experiences reported included the fear of becoming infected or infecting family members, frustration and boredom due to not being able to participate in daily activities, loss of usual routine and social isolation. Compared with participants living with other people, participants living alone reported experiencing less well-being, more anxiety and more sleep problems. They reported being sad and bored more often. There were no significant differences between the intervention and control groups in any sociodemographic variables, health status variables, or other variables associated with COVID-19. Similarly, there were no differences in terms of health management, mental health, well-being and sleep problems. Respondents with TV-AssistDem performed more memory exercises than control participants
Mosca et al. ^11^ (2020)	Italy Web of Science *Frontiers in Neurology*	1B	To describe the feasibility, adherence and appreciation of the GOAL Tele-R system (telerehabilitation)	Randomized clinical trial	Women had a statistically significant dropout rate from Tele-R activities (66%) compared to men (27%). The overall mean rehabilitation adherence (SD) score for the treated group was 84%. Participants showed high adherence to the proposed activities: a rate of 85% for the cognitive module and 83% for the physical activity module. Only one participant carried out the entire social module, and the other participants did not carry out any of the activities proposed in this module. Regarding the results of the ad hoc satisfaction questionnaire, all participants found the program useful and their mean level of appreciation of the treatment was good. In total, 92% of participants reported being satisfied with the variety of exercises and 84% gave positive feedback in terms of ease of use. A total of 76% of the patients reported a subjectively perceived benefit in relation to cognition, physical well-being and emotional benefits after the 8-week program. The proposed GOAL Tele-R system showed encouraging results in terms of feasibility, adherence and value in the cohort of MCI/VCI patients. Considering the relatively low costs and easy accessibility of this e-health intervention, the GOAL Tele-R system appears to be an efficient and promising program to care for patients with MCI/VCI

ADAS-Cog: *Alzheimer’s Disease Assessment Scale - Cognitive Scale*; CST-Brazil: cognitive stimulation therapy; GOAL Tele-R: Games for Older Adults’ Active Life; MCI: mild cognitive impairment; MoCA: *Montreal Cognitive Assesment*; SD: standard deviation; TV-AssistDem: TV-based assistive integrated service to support European adults living with dementia; VCI: vascular cognitive impairment; V-iCST: virtual individual cognitive stimulation therapy; VRRS: virtual reality cognitive rehabilitation system.

Among the main results, the feasibility and acceptance of remote cognitive stimulation activities with technologies during the pandemic stand out, reflecting on future and expanded use for different realities and cultures. In addition, some articles pointed to the stabilization of the cognitive state and primary results of improvement.

The main results found in this characterization of activities were mostly aimed at older adults with or without mild to moderate cognitive impairment, with a frequency of 2 to 3 times per week and a duration, in general, of 60 minutes per activity each day (Box 2).

**Box 2:** Characterization of the techniques used in the articles included in the systematic review.

STUDY (YEAR)	PARTICIPANTS	TECHNOLOGY USED	FREQUENCY	DURATION (TIME PER ACTIVITY)	DESCRIPTION OF THE ACTIVITIES CARRIED OUT
Hui et al. ^8^ (2022)	8 older adults aged 69 to 94 years old	Computer with internet access	14 sessions 2 days per week 7 weeks of activities	Not reported	Virtual manual with various activities such as word games, reminiscence therapies, creative activities, news, activities with music, photos, images, mathematics, finance and languages
Manenti et al. ^9^ (2020)	49 older adults with MCI	Tablet	36 sessions 3 days per week 3 months	60 minutes	Telestimulation (unstructured cognitive stimulation at home). Twelve exercises designed to improve memory, visuospatial skills, attention and executive functions
Marinho et al. ^10^ (2021)	47 older adults diagnosed with dementia	Zoom video conferencing platform	14 sessions 2 days per week 7 weeks	45 minutes	Starting with group music, followed by a warm-up exercise and a main activity based on the theme of the week (e.g., food, childhood, numbers and orientation). The sessions and activities were adapted to the groups’ abilities and in order to be as inclusive as possible
Goodman-Casanova et al. ^12^ (2020)	100 older adults aged over 60 years with cognitive impairment	Computer TV-AssistDem	The study only evaluated the feasibility/effectiveness of television-based assistive technology	Does not apply	Cognition was stimulated with stimulus memory games. Other activities were physical, social contact and informative
Mosca et al. ^11^ (2020)	31 older adults with MCI and VCI	Tablet Telerehabilitation program: GOAL-App	24 sessions 3 days per week 8 weeks	120 minutes	The cognitive module had integrated BrainHQ’s cognitive exercises. It also had a physical module and a caregiver module

MCI: mild cognitive impairment; TV-AssistDem: TV-based assistive integrated service to support European adults living with dementia; VCI: vascular cognitive impairment.

The technologies used most often in the activities were computers with internet access and tablet computers with a preinstalled program. The activities were diverse, always aimed at cognitive stimulation, and had a focus on the following domains: memory, language, attention, and praxis. They included games, mathematical calculations, images, videos, and music. In addition, they stimulated socialization via group activities and videoconferencing (Box 2).

## Discussion

The entire world population suffered enormous losses as a result of the COVID-19 pandemic. From 2020 to 2022, several repercussions of this event changed the way we live and coexist in society ^7^.

Older adults were considered a risk group for COVID-19 because they were at direct risk of developing severe illness caused by the SARS-CoV-2. Because of this, social isolation, which started as a quarantine for the entire population working in non-essential services, lasted for almost two years for the older adult population, having a major physical, cognitive and emotional impact on them. Activities that they previously carried out in person, such as consultations, weekly activity groups and socialization with family and friends, were interrupted and, due to the risk of direct contact, began to be carried out remotely with the use of technology ^7^.

According to our systematic review, four studies used a specific preintervention training technique for employing the chosen technology. The accessibility techniques used in the studies were training guided by specialists, kits with a preinstalled program and individual training, user guides with step-by-step instructions and assistance for carrying out the activities that used to be conducted by trained caregivers ^8^ ^,^ ^9^ ^,^ ^10^ ^,^ ^11^.

Remote cognitive stimulation activities have benefits, such as being accessible to all users when using low-cost technology, as demonstrated in studies using zoom videoconferencing and integrated television-based assistive technology ^10^ ^,^ ^12^.

A study from Hong Kong ^8^, carried out by psychology professionals, had as its objective and result the feasibility of adapting virtual therapy for individual cognitive stimulation in China, where changes were made to the local cultural reality, using strategies such as reminiscence activities and tools such as Chinese childhood toys, obtaining as a positive result the acceptance of activities by both family members and participants, in addition to observing improvements in the cognition, quality of life and socialization of older adults.

In a study carried out in Switzerland ^9^, neuropsychology professionals compared the use of virtual reality for cognitive stimulation with the usual face-to-face cognitive stimulation, then compared these two treatment modalities with unstructured cognitive activities at home. The group of patients that used virtual reality showed improvements in memory, language and visual-constructive skills when compared to the group that underwent the usual face-to-face treatment. It was also observed that, because it was structured, the activity with virtual reality stood out in terms of cognitive responses.

The study in Brazil ^10^ was carried out with professionals in psychology, biomedicine and psychiatry and involved an intervention based on virtual cognitive stimulation therapy using a videoconferencing platform to apply activities. The evidence indicated that the protocol and structure were feasible and brought positive results in relation to depressive symptoms, showing an improvement in the intention to treat, in addition to promoting a reduction in the burden of caregivers of participants with a lower level of education over time.

With a focus on health care, studies such as those carried out in China and Brazil involved cheap activities and do not require specific devices, using the smartphones or computers that the older adults already had and involving facilitators that directed these remote workshops according to the needs of each population. Thus, these activities were adapted to the local culture and limitations of the specific populations, demonstrating that remote workshops are practical and promote cognitive stimulation, whether aimed at prevention and health promotion or telerehabilitation ^8^ ^,^ ^10^.

Aging is active, continuous and inconvertible, due to biological, psychological and social aspects, which differ from one person to another. Its particularity is that it is multidirectional, that is, it includes gains (growth) and losses (decline), which means that individuals who enhance gains and reduce losses tend towards active aging ^13^.

Dementia can cause a progressive disorder of multiple cognitive functions, such as memory, attention, learning, calculation, comprehension, orientation, thinking, language and judgment, causing sensory deficits that can lead to problems in the way older adults process environmental stimuli, impairing sensory functioning and cognitive response. For this reason, it is essential to determine these individuals’ functional capacity and to help give new meaning to their lives, especially when they already have a disease. Such actions prevent the negative repercussions of comorbidities, allowing the development of more appropriate care planning and helping the older person to have a full life and participate socially in it ^3^ ^,^ ^14^.

A study in Brazil ^15^ analyzed the national scenario of health promotion programs and prevention of risks and diseases for older people from 1999 to 2019. During that period, nurses accounted for 93% of the professionals working in the field. The results of the analysis showed that there were 87 programs aimed at the older population, more than 50% of which focused on chronic comorbidities and healthy eating. But cognitive stimulation and/or telerehabilitation programs were still scarce.

A study found that cognitive improvements appear from six months to five years after older adults start to participate in memory stimulation activities ^16^. The authors also discussed the need for changes to expand and transform the current care model in order to achieve the goals of physical and mental independence for older adults ^16^. For this reason, it is necessary to renew health promotion models so that a greater number of individuals can engage in these activities ^15^.

At the Madrid Conference (2002), the United Nations established the importance of reciprocity between generations and of intergenerational actions, highlighting the encouragement of social interaction among the older people and the fight against prejudice, which are fundamental to transform the social perception of aging as a sign of disability and incapacity ^17^. To this end, it is essential to recognize the value of coexistence between various age groups and multidisciplinary health teams. The technology that is associated with younger generations can be a strong ally for these actions and for expanding health promotion actions.

According to the Brazilian Institute of Geography and Statistics (IBGE, acronym in Portuguese) ^18^, every year there is an increase in internet access and the use of technology by older adults, even though culturally they are the most digitally excluded population group. From 2019 to 2021, the number of older individuals who frequently used the internet increased from 44.8% to 57.5% ^18^ ^,^ ^19^.

The survey carried out by the Brazilian National Confederation of Store Managers (CNDL, acronym in Portuguese) in partnership with the Credit Protection Service (SPC) indicated that 97% of older Brazilian adults access the internet, also showing that the number of elderly people who access the internet grew from 68% in 2018 to 97% in 2021 ^20^.

Due to the constant growth in the older population, which generates economic and sociocultural challenges, meeting each person’s needs is an obstacle to be overcome. Due to the COVID-19 pandemic, these barriers have become greater and the difficulties have been amplified. This is attributed to the social isolation and restriction measures for older adults, who are considered the most affected population and who are at highest risk of developing severe COVID-19 ^21^.

During the COVID-19 pandemic, most therapeutic cognitive stimulation workshops for older adults - consultations that are usually carried out by health professionals face-to-face and are essential for the physical and mental health of older adults, as well as for prevention and health promotion - started to be carried out remotely. Due to this situation, several activities had to be reformulated and adapted to the new reality. As a consequence, there was an increase in social isolation, cognitive decline and feelings of loneliness ^22^.

Information and communication technologies (ICT), such as cell phones, smartphones and computers, were used as tools to bring people together, socialize and return to health activities, as well as to promote cognitive and motor stimulation in older adults ^22^. In the health sector, ICTs were mainly used in promotion and prevention to help solve the problem of social distancing.

Research shows that remote workshops, telehealth and telerehabilitation for the older population have positive results, such as improvement in cognitive aspects via pre- and post-intervention testing; and evidence shows that the access to devices such as smartphones, tablets and computers facilitates communication and connection, reducing feelings of isolation and loneliness ^23^ ^,^ ^24^ ^,^ ^25^.

A systematic review of digital health interventions ^26^, based on 20 studies, showed that digital health interventions produced a moderate effect on the performance of cognitive skills of the intervention group, which was composed of participants who had mild cognitive impairment, compared to the control group, which was composed of participants who had the same cognitive conditions.

The acceptance of this type of activity was reported in several studies that used technology for cognitive stimulation, such as the one carried out in Italy, which showed encouraging results regarding the feasibility of cognitive telerehabilitation activities, as well as participants’ adherence to and appreciation of them ^11^. In the study carried out in China ^8^, participants expressed that they enjoyed the activities and the fact that there was virtual facilitated participation.

There are few studies on the use of remote cognitive stimulation activities. Few studies were found with the specific objective of describing the frequency, duration and structural methodology of cognitive activities applied in this virtual form, making it difficult to fully replicate these practices in other locations, leading researchers interested in applying a remote cognitive stimulation activity to have to elaborate the entire structure and its methodology.

## Conclusion

In the remote cognitive stimulation activities for older adults carried out during the COVID-19 pandemic and described by the five selected articles, a mean of 47 individuals took part, the technologies used were tablet and personal computers, preinstalled programs were the most used strategy, interventions lasted from one to three months, and activities were conducted 2 to 3 times per week. These remote therapies proved to be viable and accepted by the older adults, showing positive results via tests and the participants’ testimonies.

It is necessary to continue these notoriously important studies, both during the pandemic, to minimize the impacts of social isolation, and for the possible application of remote cognitive stimulation activities, to achieve equity in this practice of prevention, protection and health promotion and make it accessible for all older adults.

As a follow-up proposal to this study, it is intended to carry out an intervention study describing the activities of virtual workshops for cognitive stimulation and comparing their participants to those of a follow-up group that did not take part in these activities.
